# ROCS: Receiver Operating Characteristic Surface for Class-Skewed High-Throughput Data

**DOI:** 10.1371/journal.pone.0040598

**Published:** 2012-07-06

**Authors:** Tianwei Yu

**Affiliations:** Department of Biostatistics and Bioinformatics, Rollins School of Public Health, Emory University, Atlanta, Georgia, United States of America; University of Iowa, United States of America

## Abstract

The receiver operating characteristic (ROC) curve is an important tool to gauge the performance of classifiers. In certain situations of high-throughput data analysis, the data is heavily class-skewed, i.e. most features tested belong to the true negative class. In such cases, only a small portion of the ROC curve is relevant in practical terms, rendering the ROC curve and its area under the curve (AUC) insufficient for the purpose of judging classifier performance. Here we define an ROC surface (ROCS) using true positive rate (TPR), false positive rate (FPR), and true discovery rate (TDR). The ROC surface, together with the associated quantities, volume under the surface (VUS) and FDR-controlled area under the ROC curve (FCAUC), provide a useful approach for gauging classifier performance on class-skewed high-throughput data. The implementation as an R package is available at http://userwww.service.emory.edu/~tyu8/ROCS/.

## Introduction

Receiver Operating Characteristic (ROC) curve is widely used to assess the performance of classifiers in biomedical research [1], [2], [3]. The curve is generated by plotting the true positive rate (TPR) against the false positive rate (FPR). Generally speaking, it depicts the level of separation between two distributions, one corresponding to the true negatives, and the other corresponding to the true positives, given the scores from a classifier [4]. The area under the curve (AUC) summarizes the main characteristic of the ROC with a single number, greatly facilitating methods comparison [4].

In many applications on high-throughput data, the number of true positives is magnitudes smaller than the number of true negatives. Examples include analyzing high-throughput data with spike-in gold standards [5], detecting break points of copy numbers [6], and many other situations where only top-ranked features are of interest [7]. In such situations, at a very low false positive level, the false discovery rate (FDR) [8] could become unacceptably high. Thus only a small corner of the ROC curve is relevant in practical terms, and the AUC can no longer summarize the effectiveness of the separation. To address this issue, some methods were developed to focus on sub-regions of the ROC curve, either by computing partial area under the curve [9], or by magnifying portions of the ROC smoothly [7]. Another heuristic approach was to use a variant of ROC – replacing the false positive rate (FPR) with the false discovery rate (FDR) in the ROC plot [5], [6], [10]. Unlike the FPR and the TPR, the FDR may not be a monotone function of the model parameter(s) controlling sensitivity. When the data is class-skewed, two main factors influence the capability to detect positive features effectively – the level of separation between the two distributions, and the class ratio, i.e. the ratio between the number of true negatives and the number of true positives. None of the aforementioned methods convey both pieces of information together effectively.

Here we describe a method and the associated R package that depict the relationship using a three-dimensional surface based on TPR, FPR, and the true discovery rate (TDR), which is one minus FDR. Because the TPR-FPR-TDR data forms a curve instead of a surface in the three-dimensional space, defining an ROC surface is not straight-forward. In this manuscript we combine the ideas of the original ROC and its FDR-TPR variant. We show that such an ROC surface enjoys intuitive interpretation, as well as loyally reflects class separation in the class-skewed scenario. Two related quantities, the volume under the surface (VUS) of the ROC surface and the FDR-controlled area under the curve (FCAUC), as well as a testing procedure, are also defined to help simplify comparison of classifiers in the class-skewed scenarios.

## Methods

### ROC Surface (ROCS) and its Volume Under the Surface (VUS)

**Figure 1:**
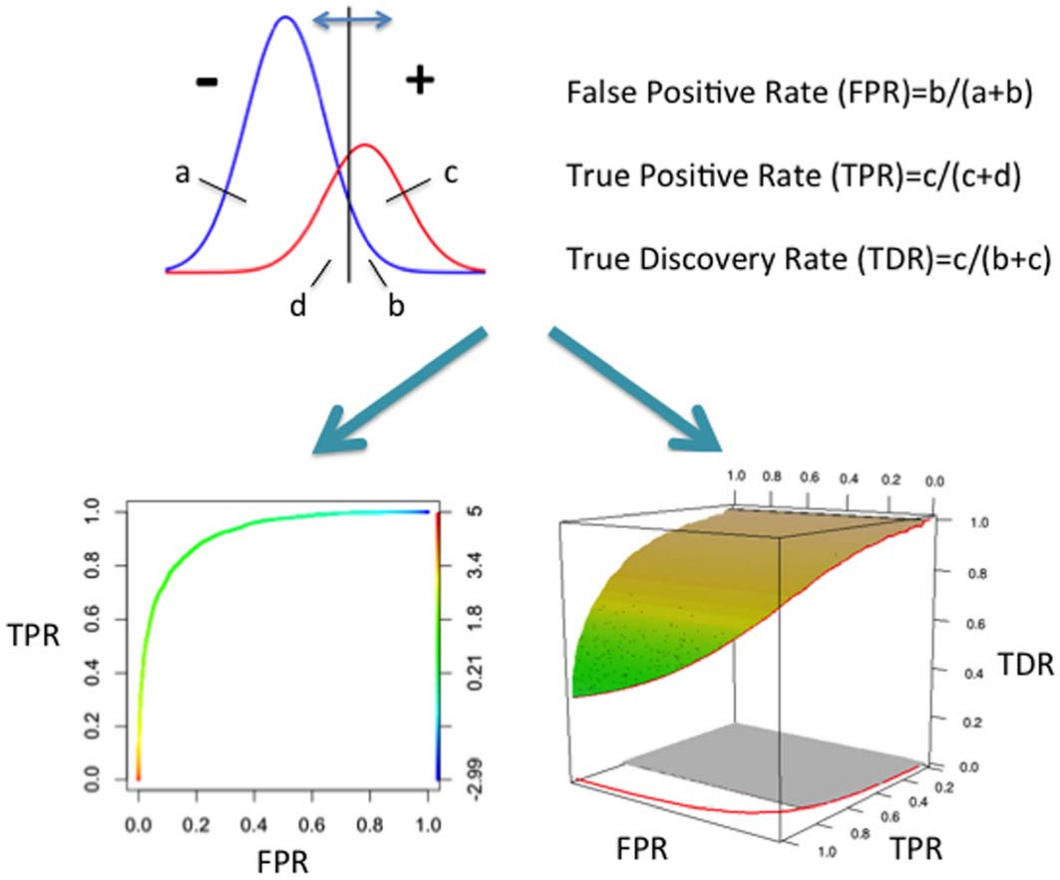
Illustration of the construction of the ROC curve and the ROC surface (ROCS).

Generally speaking, a high-throughput dataset contains multi-dimensional measurements for each feature under study, e.g. genes, metabolites etc. When a certain model is applied, it reduces the measurement on each feature to a scalar. Given that each feature belongs to either the true positive or the true negative group, changing the threshold value *δ* will generate a tradeoff between sensitivity and specificity, hence the ROC curve (Fig. 1). Here we use *δ* only conceptually. It may represent a threshold on the reduced data, or certain parameter(s) in the model that controls sensitivity. The *TPR, FPR, TDR* are all functions of *δ*: *TPR = TPR(δ), FPR = FPR(δ),* and *TDR = TDR(δ).* The traditional AUC of ROC (Fig. 1) is defined as
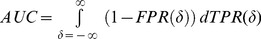
(1)


We define the ROC surface by the surface formed between the FPR-TPR-TDR curve and its projection on the TPR-TDR plain (Fig. 1). The projection of the surface on the FPR-TPR plain is the area under the regular ROC curve. The volume between the ROC surface and its projection on the FPR-TPR plain is defined as the volume under the ROC surface (VUS), 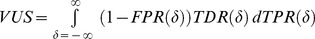
(2)


Compared to equation (1), we see this VUS can be viewed as a weighted version of the AUC of the regular ROC curve, with the weight at every TPR level being the corresponding TDR values. This definition makes intuitive sense because it gives higher weight to higher TDR regions. In real data analysis, the curves are approximated by step functions, and integrations are replaced by summations.

The maximum possible value of the VUS is one, which is achieved when the true positive and true negative samples can be perfectly separated, no matter what the class ratio is. The minimum value of the VUS approaches zero in the scenario that the two distributions are inseparable, and the class ratio (# true negatives/# true positives) approaches infinity.

### Assessing the Significance of the Class Separation

The statistical significance of the between-class separation can be assessed by a permutation test in which the null distribution of the VUS value is generated by permuting class labels of the samples. We randomly permute the true positive/true negative labels *K* times and compute the VUS values 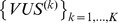. The proportion of the sampled permutations with VUS values larger than the observed value is taken as the p-value of the one-sided test,
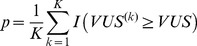
(3)where *I(A)* is the indicator function which is 1 if *A* is true and 0 otherwise.

**Figure 2:**
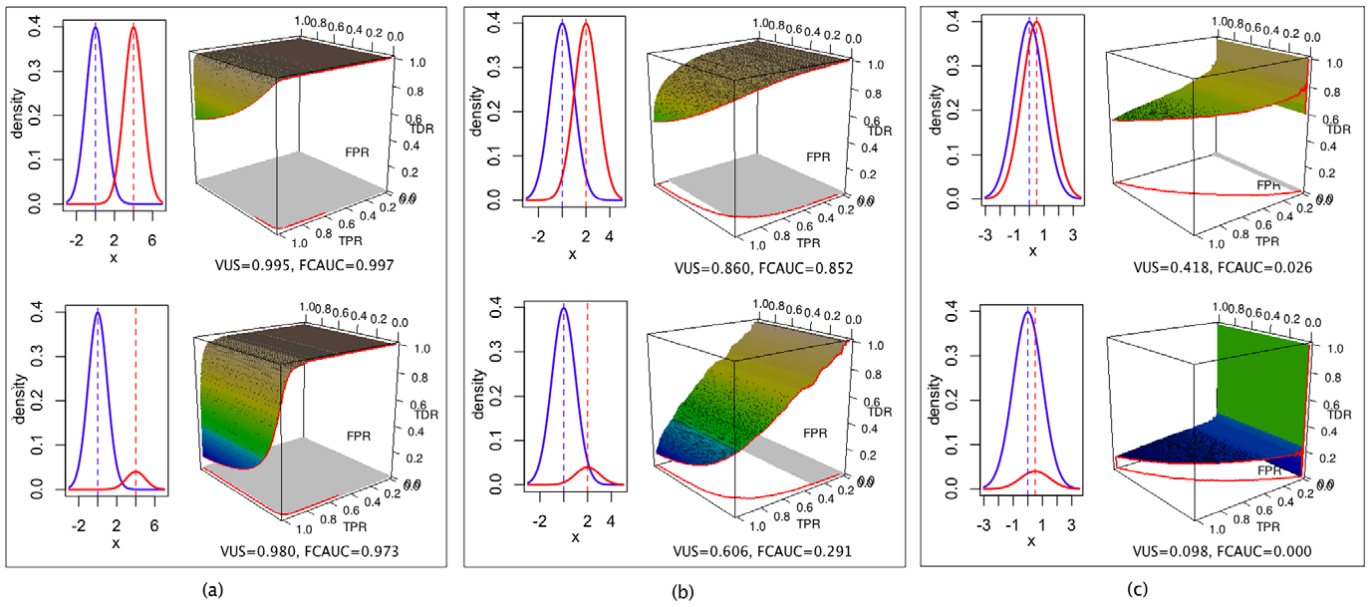
Illustration of the ROCS under different scenarios. Within each box, the ROCS plot (right) was generated from 10000 random samples drawn from the two Gaussian distributions (left). Upper panel: class ratio negative:positive = 1∶1; lower panel: class ratio negative:positive = 10∶1. (a) Two well-separated distributions; (b) two moderately separated distributions; (c) two severely overlapping distributions. Blue: true negative class; red: true positive class. Shaded area in the TPR-FPR plain: region under the ROC curve corresponding to FDR equal to or lower than 0.2.

**Figure 3:**
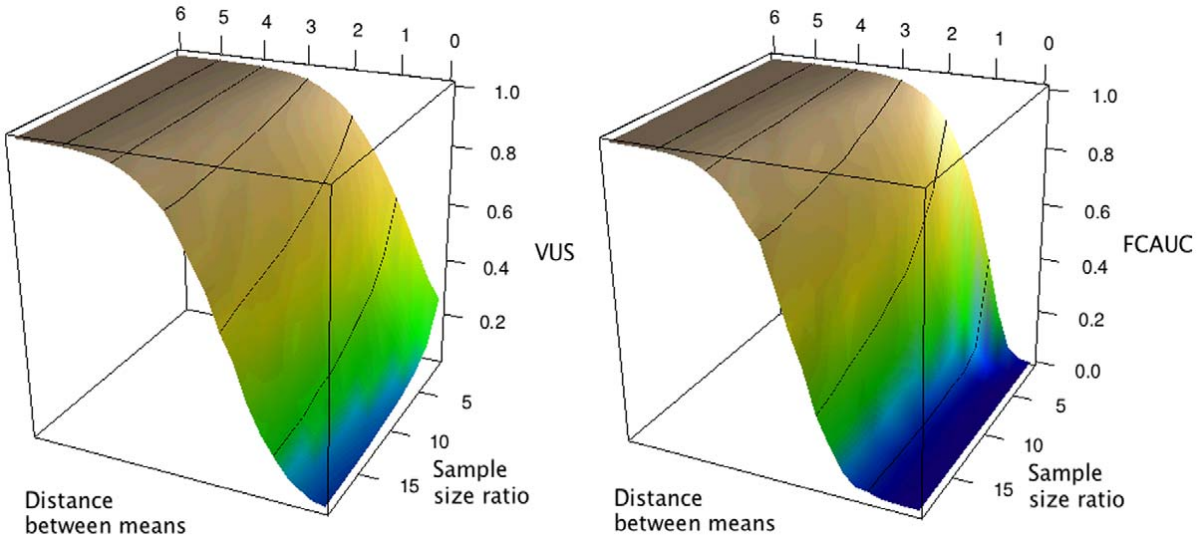
Simulation results – average VUS and FCAUC values as functions of the distance between the mean of the true negative and true positive distributions and the class ratio (#negative/#positive). The two distributions are both normal with standard deviation one. Black curves: values corresponding to integer distances between means.

#### Testing for significant difference between two ROCS

The null hypothesis is the two studies are generated from the same class-specific distributions of ranks. It implies 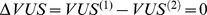 when the two studies have the same class ratios. For both studies, we ensure the median of the true positive class is to the right of the true negative class by multiplying the measurements with *−1* when necessary. Let the first ROCS be constructed from *n_1_* true positive samples and *n_0_* true negative samples. Let the second ROCS be constructed from *m_1_* true positive samples and *m_0_* true negative samples.

1. In order to establish the distribution under the null hypothesis, we remove the impact of difference in class ratios (true negatives v.s. true positives) between the two studies by re-sampling. From each study, we re-sample *(n_0_+m_0_)/2* true negatives and *(n_1_+m_1_)/2* true positives. When the two values are not integer, they are rounded to the closest integer.
2. Within each study, we transform the new samples into ranks. For tied measurements, we assign random ranks.
3. We merge the ranks of the true negative samples from both studies to estimate the true negative distribution under the null hypothesis, and merge the ranks of the true positive samples from both studies to estimate the true positive distribution under the null hypothesis.
4. We take *B* samples with replacement from the constructed distributions under the null. Each sample contains *n_1_* true positives and *n_0_* true negatives for study 1, and *m_1_* true positives and *m_0_* true negatives for study 2. We compute the VUS value of the ROCS, 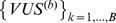. The p-value of the test is
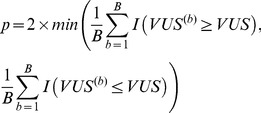
(4)where *I(A)* is the indicator function which is 1 if A is true and 0 otherwise

**Figure 4:**
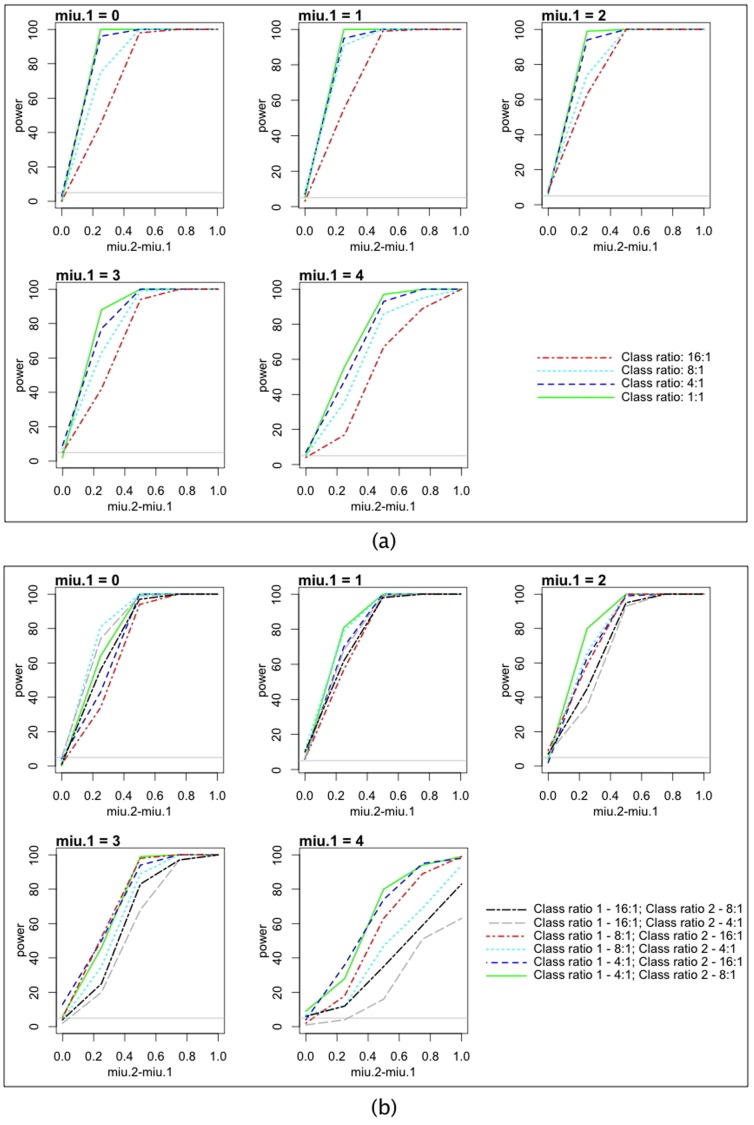
Simulation results on the power and size of the test. **In all simulations, the true negative class mean is set at zero for both studies, and the standard deviations of the true negative class and the true positive class are one for both studies.** Miu.1 is the true positive class mean of study 1; miu.2 is the true positive class mean of study 2. All curves are based on 100 simulations. Horizontal line: the alpha level 0.05 for the purpose of examining the size of the test. (a) The simple scenario where both studies have the same sample size (4000) and class ratio. (b) The more complex case where study 1 has 2000 samples, study 2 has 4000 samples, and the class ratios are different.

### FDR-Controlled Area Under the Curve (FCAUC)

We define FCAUC as the area under the traditional ROC curve corresponding to a pre-determined FDR threshold. Let *b* be the cutoff of FDR. Assuming the distribution of the true positive class is to the right of the true negative class, we find the minimum cutoff that corresponds to the acceptable FDR level: 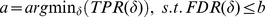



Then the FCAUC value is found by,
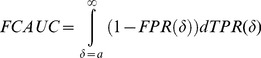
(5)


Similar to the VUS, the significance of the FCAUC can be assessed by permutation test. We randomly permute the true positive/true negative labels *K* times and compute the FCAUC values 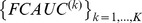. The proportion of the sampled permutations with FCAUC values larger than the observed value is taken as the p-value of the one-sided test,
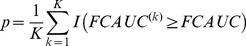
(6)where *I(A)* is the indicator function which is 1 if *A* is true and 0 otherwise.

## Results and Discussion

### Demonstration of the ROCS Utility using Simulated Data

First we illustrate the behavior of the ROCS using a few examples. We vary the level of overlap between the true negative and true positive distributions, and the ratio between true negative and true positive sample counts (Fig. 2). When the distributions of the true negative class and true positive class are well separated (Fig. 2a), the VUS and the FCAUC values are close to one, both at the class ratio of 1∶1 and 10∶1. When the two distributions partially overlap (Fig. 2b), the VUS and FCAUC values decrease with the proportion of true positive samples. This is because the TDR levels become lower when the proportion of true positive samples decreases, even though the TPR-FPR curve doesn’t change. When the two distributions severely overlap (Fig. 2c), the VUS and FCAUC values are further decreased. When the class ratio is large, the VUS value becomes close to zero, and the FCAUC becomes zero because at no cutoff level does the FDR gets below 0.2. In all three scenarios, the traditional ROC curves are the same between the upper and lower plots because the true negative and true positive distributions do not change.

**Figure 5:**
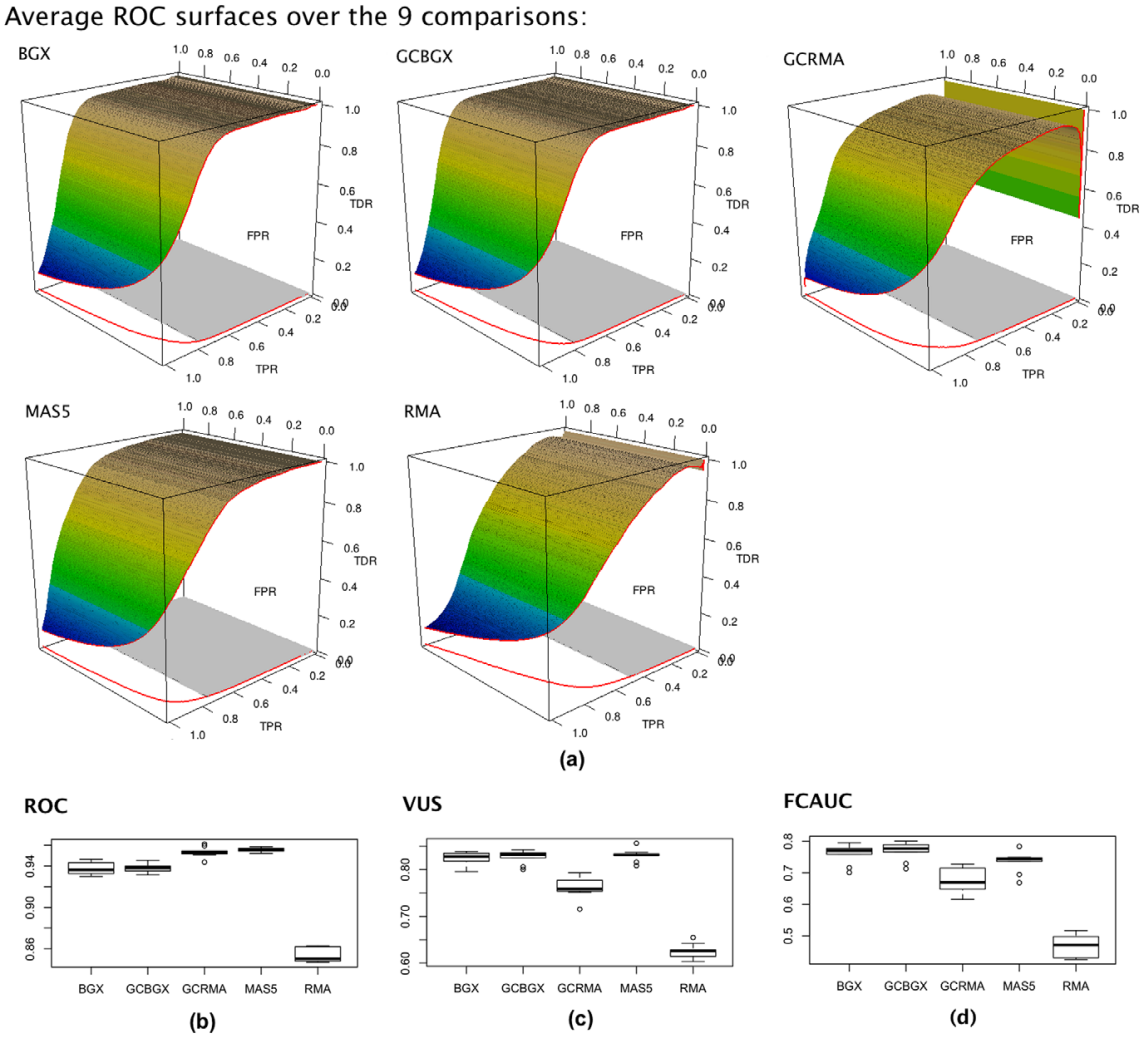
Illustration of the ROCS procedure using the results on the golden spike dataset [**5**] by Turro et al [**10**]. (a) ROCS plots of the results. (b) Comparing the regular AUC under ROC of the nine single array pairs. (c) Comparing the VUS values of the nine single array pairs. (d) Comparing the FCAUC values of the nine single array pairs. Please note that the nine comparisons in Turro et al [10] are not independent. Thus the distributions in (b), (c) and (d) only serve to illustrate the values. They do not have any implications of significant differences between methods.

We then systematically examine the relationship between VUS value and the distance between the mean of the true negative and true positive distributions, as well as the class ratio (number of true negatives versus number of true positives). We generate data from normal distributions with standard deviation of one. Different distances between the two means and different class ratios were simulated. At each parameter setting, we repeated the simulation 50 times, each with 2000 samples, and took the mean VUS value. When the mean values of the two distributions are far apart, the VUS value approaches one, regardless of the class ratio (Fig. 3, left panel). When the overlap is moderate to high, the VUS values decrease with the increase of class ratio (*# true negatives/# true positives*). The VUS value approaches zero when the two distributions are identical and the class ratio is large (Fig. 3, left panel). The same simulation procedure was applied to the FCAUC value. Its behavior was very close to that of the VUS, except when the two distributions are close to identical, the FCAUC value becomes zero at all class ratio settings (Fig. 3, right panel).

**Figure 6:**
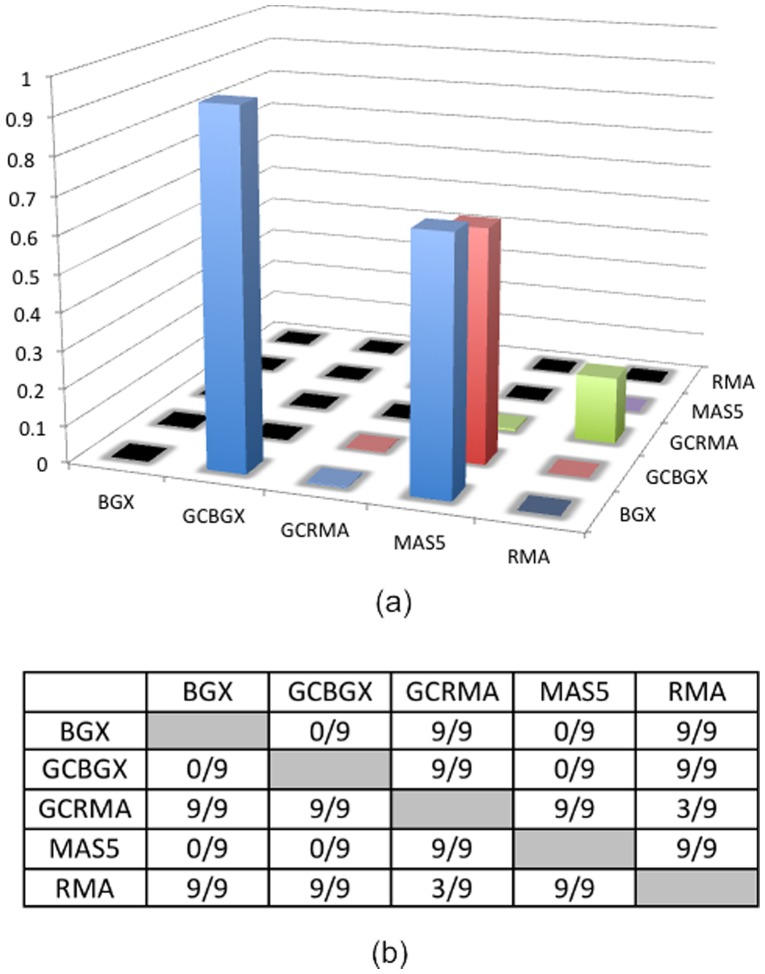
Testing the difference in VUS between different methods. (a) Average p-values over the nine comparisons. (b) Fractions of the nine comparisons being significant (p-value <0.05). Please note that the nine comparisons in Turro et al [10] are not independent.

**Figure 7:**
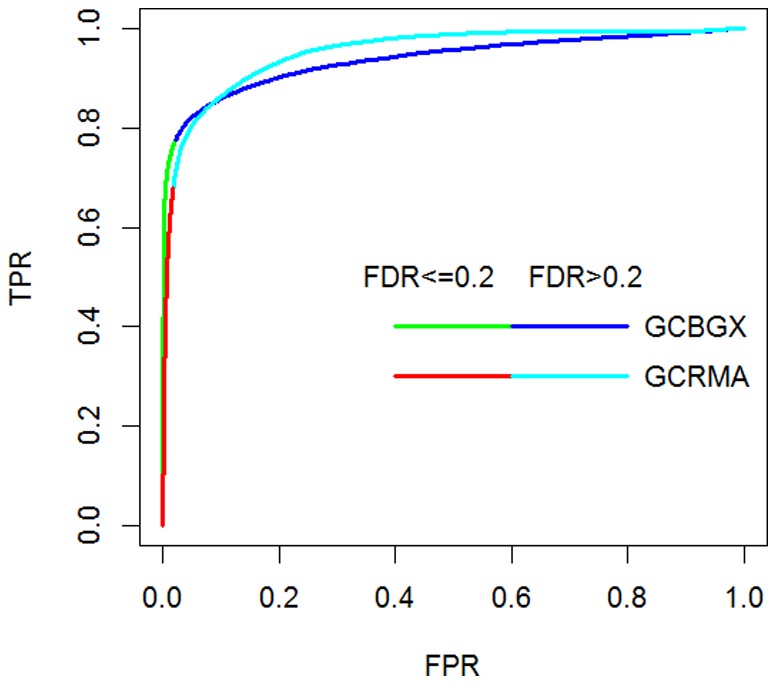
Comparing the ROC curves between the methods GCBGX and GCRMA. The values were averaged over the nine comparisons. Colors in each curve represent FDR levels dichotomized at the cutoff value of 0.2.

A simulation study was conducted to assess the behavior of the testing procedure between ROC surfaces. In every simulation, we generated true-negative and true-positive samples for two fake studies. For both fake studies, the true-negative class follows the standard normal distribution, and the true-positive class follows normal distributions with standard deviation of one with varying mean values. In the first set of simulations, both studies have the same sample size and class ratio. In the situations where the two mean values of the true positive class are the same (the null hypothesis is true), the test has ∼5% error rate, which indicates correct size of the test (Fig. 4a). When the difference between the mean values deviates from zero, the power to detect the difference increases quickly. The statistical power to detect the differences is also negatively associated with the class ratio, with 1∶1 ratio yielding the highest power, and 16∶1 yielding the lowest (Fig. 4a). In the second set of simulations, we used different sample sizes (2000 and 4000) for the two studies, and allowed the class ratio to be different between the two studies. The tests maintained the correct size in such settings (Fig. 4b). Again the power rises quickly with the differences between the true positive class means (Fig. 4b).

### Demonstration of the ROCS Utility using Real Data

The purpose of the demonstration here is not comparing the methods. Rather, it is to show that the ROCS is useful in practice for visually assessing the differences between methods, and the VUS and FCAUC values are convenient and relevant measures for performance assessment.

We use the analytical results by Turro et al [10], in which the authors compared the performance of their microarray data analysis method BGX with some popular methods using the golden spike dataset [5]. The golden spike dataset was generated using spike-in experiment. It contains 14010 genes, in which 1331 were differentially expressed by spiking in [5]. Turro et al made nine single-array comparisons using five methods between the two experimental conditions (spiked and control), and presented the results with ROC curves averaged over the nine comparisons using the FDR-TPR variant of ROC [10]. Although the curves visually showed some differences between methods, they did not offer a measure to quantify the difference, and did not convey the level of variation between the nine comparisons.

Using the analytical results provided on the website of the Turro paper [10], we carried out data presentation and analysis with ROCS. By generating ROC surface plots (Fig. 5a), ROCS analysis conveys the FPR dimension of the difference, which was not present in figure 8 of [10]. One interesting new observation is that, although GCRMA clearly has a lower ROC surface than BGX, GCBGX and MAS5 (Fig. 5a), in the traditional ROC dimension, i.e. the FPR/TPR space, its ROC curve is similar to those of BGX, GCBGX and MAS5 (Fig. 5a). Indeed the AUC of ROC from GCRMA is on-par with the three methods BGX, GCBGX and MAS5 (Fig. 5b). However, because of its inferior performance in the FDR domain, its ROC surface is lower than BGX, GCBGX and MAS5 (Fig. 5a), which is reflected in lower VUS and FCAUC values (Fig. 5c, 5d).

In each of the nine comparisons, the relative rankings of genes were given by each of the methods. Using the rankings, we conducted testing between the five methods regarding their difference in ROCS. The testing was done nine times between each pair of methods. Figure 6 shows the average p-values between each pair of methods (Fig. 6a), as well as the number of times the testing result was significant at the alpha level of 0.05 (Fig. 6b). It clearly showed that BGX, GCBGX and MAS5 results are similar, while GCRMA and RMA results are somewhat similar. There are substantial differences between the two groups (Fig. 6).

ROC and AUC alone do not show much difference between GCRMA versus the group of BGX, GCBGX and MAS5 (Fig. 5b), yet ROCS analysis and VUS/FCAUC show substantial differences (Fig. 5c & 5d). To explore the reason, we compare GCRMA and GCBGX (Fig. 7). The two ROC curves have similar shape. In fact, the GCRMA curve has higher AUC overall. However, under heavy class skew, only the left-most portion of the two curves are meaningful in practical terms. The two curves differ a lot in that region. At the FDR cutoff of 0.2, GCBGX yields 15% more positive genes than GCRMA (1285 versus 1117). This is a substantial difference that traditional ROC cannot reveal. Overall, ROCS analysis reveals more information than traditional ROC analysis, and provides more quantifiable results than simply using the FDR-TPR variant of ROC plot as in [10].

### Implementation

The method is implemented as an R package available at http://userwww.service.emory.edu/~tyu8/ROCS/. The package includes functions of generating ROCS plots and VUS/FCAUC from raw data, or from user-provided FPR, TPR and FDR. The package produces rotatable three dimensional plots using the 3D real-time visualization device (package RGL) in R [11]. It contains the testing procedures described in the Methods section. The package also incudes functions that generate traditional ROC plots in which the area corresponding to acceptable FDR level is colored, both from raw data and from user-provided FPR, TPR and FDR values. Unlike traditional ROC analysis, when using the ROCS analysis, true negative and true positive class labels are not exchangeable.

In summary, we developed a new approach to incorporate FDR information into the ROC analysis. This approach addresses the issue of severe class-skewness in ROC analysis by explicitly utilizing the inference of false discovery rate. The ROCS plot and the associated VUS and FCAUC values are useful tools for judging classifier performance when true negatives greatly out-number true positives in high-throughput data. We shall note that unlike the ROC curve, the ROC surface and its associated quantities are dependent upon the class ratio. The results are meaningful only when the class ratio of the training data is close to that of future data. In the case that the user foresees a discrepancy, the issue can be addressed by (repeated) resampling from the training data to achieve targeted class ratios and building ROCS.
